# Non-obesogenic doses of palmitate disrupt circadian metabolism in adipocytes

**DOI:** 10.1080/21623945.2019.1698791

**Published:** 2019-12-03

**Authors:** Yael Tal, Nava Chapnik, Oren Froy

**Affiliations:** Institute of Biochemistry, Food Science and Nutrition, Robert H. Smith Faculty of Agriculture, Food and Environment, The Hebrew University of Jerusalem, Rehovot, Israel

**Keywords:** Palmitate, metabolism, adipocyte, clock, circadian

## Abstract

Saturated fatty acids, such as palmitate, lead to circadian disruption. We aimed at studying the effect of low doses of palmitate on circadian metabolism and to decipher the mechanism by which fatty acids convey their effect in adipocytes. Mice were fed non-obesogenic doses of palm or olive oil and adipocytes were treated with palmitate and oleate. Cultured adipocytes treated with oleate showed increased AMPK activity and induced the expression of mitochondrial genes indicating increased fatty acid oxidation, while palmitate increased ACC activity and induced the expression of lipogenic genes, indicating increased fatty acid synthesis. Low doses of palmitate were sufficient to alter circadian rhythms, due to changes in the expression and/or activity of key metabolic proteins including GSK3β and AKT. Palmitate-induced AKT and GSK3β activation led to the phosphorylation of BMAL1 that resulted in low levels as well as high amplitude of circadian clock expression. In adipocytes, the detrimental metabolic alteration of palmitate manifests itself early on even at non-obesogenic levels. This is accompanied by modulating BMAL1 expression and phosphorylation levels, which lead to dampened clock gene expression.

## 1. Introduction

The circadian clock, located in the suprachiasmatic nuclei (SCN) of the anterior hypothalamus, responds to the environmental light–dark cycle and generates endogenous rhythms of ~24 h [1]. Similar clock oscillators have been found in peripheral tissues, such as the liver, intestine and adipose tissue [2], regulating cellular and physiological functions [1,3,4]. The core clock mechanism in both SCN neurons and peripheral tissues is encoded by the genes *Clock* and *Bmal1* (brain–muscle-Arnt-like 1) that heterodimerize and bind to E-box sequences to mediate transcription of a large number of genes, including the *Periods* (*Per1, Per2, Per3*) and *Cryptochromes* (Cry1, Cry2). PERs and CRYs constitute part of the negative feedback loop that inhibits CLOCK:BMAL1-mediated transcription [1]. Disruption of the coordination between the endogenous clock and the environment leads to attenuated diurnal feeding rhythms, hyperphagia and obesity [5–8].

The circadian clock regulates metabolism and energy homoeostasis in adipose tissues [9]. This is achieved by mediating the expression and/or activity of certain metabolic enzymes and transport systems [10]. In addition to its role in the core clock mechanism, BMAL1 is highly induced during adipogenesis and was found to regulate adipogenesis and adipocyte function [11,12]. Moreover, RORα, the positive regulator of *Bmal1* expression [13] has been shown to regulate lipogenesis and lipid storage in skeletal muscle [4]. Deficiency in REV-ERBα, the negative regulator of BMAL1, elevates lipoprotein lipase levels in peripheral tissues including liver, muscle and adipose tissue, correlating with increases in body weight and overall adiposity [14]. In skeletal muscle, REV-ERBα deficiency led to reduced mitochondrial content and oxidative function resulting in compromised exercise capacity, while overexpression and pharmacological activation of the receptor led to an improvement [15]. Peroxisome proliferator-activated receptor gamma (PPARγ) was demonstrated to directly regulate the circadian expression of *Bmal1* [16] and circadian behaviour and metabolism [17]. Moreover, the transcriptional activity of PPARγ was found to be repressed through interaction with PER2, and, as a result, *Per2* null mice show altered lipid metabolism [18]. Glycogen synthase kinase 3 beta (GSK3β) has been shown to phosphorylate most clock proteins, thereby controlling their stability and subcellular localization [19]. Specifically, GSK3β phosphorylates BMAL1, an event that controls the stability of the protein and the amplitude of circadian oscillation. BMAL1 phosphorylation appears to be an important regulatory step in maintaining the robustness of the circadian clock [20].

High-fat diet (HFD) has been shown to influence clock oscillation and function in various animal studies [21–24]. These findings hint towards the possible involvement of lipids in circadian control [25]. It was shown that 3 days of a high-fat diet were sufficient to impose reprogramming of the circadian clock [26]. The rapid influence of the diet on the clock suggests that the nutritional challenge, and possibly lipids themselves, and not merely the development of obesity, are sufficient to alter clock function.

The saturated fatty acid palmitate (C16:0) and the monounsaturated fatty acid oleate (C18:1), are the most common fatty acids in human diets as well as in animal and human fat tissue. While palmitate is associated with the development of obesity, and Type 2 diabetes, lipotoxicity and oxidative stress, oleate has been shown to prevent or alleviate the toxic effect of saturated free fatty acids and to be protective against insulin resistance and metabolic disorders [27]. Palmitate has been associated with circadian dysregulation in different cell lines [28–32]. In a recent study, non-obesogenic doses of the fatty acids palmitate and oleate showed different effects on circadian rhythms and metabolism both *in vitro* in hepatocytes and *in vivo* in mouse liver. Oleate activated the AMPK–SIRT1 signalling pathway leading to inhibition of fatty acid synthesis and increased fatty acid oxidation, whereas palmitate activated mTOR signalling leading to increased fatty acid synthesis. This was achieved by modulating BMAL1 at several levels abrogating its activity and expression [33]. In this study, we aimed to elucidate the effect of low doses of oleate and palmitate on circadian metabolism in adipocytes.

## 2. Materials and methods

### 2.1. Animals, treatments and tissues

Ten-week-old C57BL/6 male mice (Harlan Laboratories, Jerusalem, Israel) were housed in a temperature- and humidity-controlled facility (23–24°C, 60% humidity). Mice were entrained to 12 h light and 12 h darkness for 2 weeks with food available *ad libitum* and then were randomly assigned to either olive oil (n = 24) or palm oil diet (n = 24) for 3 weeks. Control diet (n = 24) contained cornstarch 52.7% (w/w), casein 20% (w/w), sucrose 10% (w/w), soybean oil 7% (w/w), cellulose 5% (w/w), mineral mix 4% (w/w), vitamin mix 1% (w/w), methionine 0.3% (w/w). In palm and olive oil diet groups, soybean oil was replaced with olive oil (fatty acid composition: C:14˂0.03, C:16 18.4%, C:16.1 2.01%, C:18 3.75%, C:18–1 68.2%, C:18–2 7.6%) or palm oil (fatty acid composition: C:12 0.2%, C:14 1.1%, C:16 44.3%, C:18 4.6%, C:18–1 39%, C:18–2 10%). Body weight was recorded weekly and at the end of the experiment. Daily food intake was monitored every 2 days. Mice were placed on an overnight fast (12 h) before anaesthesia and epididymal adipose tissue was removed every 4 h (n = 4 for each time-point) around the circadian cycle in total darkness under a dim red light to avoid the masking effects of light. Tissue was immediately frozen in liquid nitrogen and stored at −80°C. Mice were humanely killed at the end of the experiment. Animals received humane care according to the criteria outlined in the ‘Guide for the Care and Use of Laboratory Animals’ prepared by the National Academy of Sciences and published by the National Institutes of Health (NIH publication 86–23 revised 1985). The joint ethics committee of the Hebrew University and Hadassah Medical Centre approved this study (approval no. AG-14-13490-3).

### 2.2. Cell culture experiments

3T3-L1 pre-adipocytes were grown in Dulbecco’s modified Eagle’s medium (DMEM) supplemented with 10% calf serum and 5% CO_2_ at 37 °C. Differentiation of pre-adipocytes to adipocytes was achieved by allowing the cells to reach confluence. Two days later, the medium was replaced with DMEM supplemented with 10% FCS, 0.5 mM 3-isobutyl-1-methylxanthine, 1 μM dexamethasone, and 5 μg/mL insulin. Every 2 days thereafter, fresh medium (DMEM plus 10% FCS and 5 μg/mL insulin) was added to the culture for 8 days. On the 8th day of differentiation, cells were synchronized with a 1-h pulse of 1 mM dexamethasone (Sigma, Rehovot, Israel) and medium was replaced with fresh DMEM supplemented with palmitate or oleate mixed in 1% bovine serum albumin (BSA) to a final concentration of 50 μM. For Control, medium was replaced with fresh DMEM containing 1% BSA. Following 6 h of incubation, cells were harvested in duplicates per treatment per time-point every 6 h for 24 h. Three independent experiments were performed with 2 replicates in each experiment.

### 2.3. Oil Red O staining

Lipid quantification in 3T3-L1 cells was performed using Oil Red O staining. Cells were fixed in 10% formaldehyde in aqueous phosphate buffer overnight, washed with 60% isopropanol and stained with Oil Red O solution (in 60% isopropanol) for 10 min. Cells were then repeatedly washed with water and destained in 100% isopropanol for 10 min. The optical density of the isopropanol solution was measured at 500 nm.

### 2.4. RNA extraction and quantitative real-time PCR

RNA of cells and tissue was extracted from cells using TRI Reagent (Sigma). Total RNA was DNase I-treated using RQ1 DNase (Promega, Madison, WI, USA) for 2 h at 37°C, as was previously described [34]. Two μg of DNase I-treated RNA were reverse transcribed using MMuLV reverse-transcriptase and random hexamers (Promega). One twentieth of the reaction was then subjected to quantitative real-time PCR using SYBR Green Supermix (Quanta Biosciences, Beverly, MA, USA) and primers **(Table S1)** spanning exon-exon boundaries and the ABI Prism 7300 Sequence Detection System (Applied Biosystems, Foster City, CA, USA). Gene expressing was normalized to actin. Reaction conditions were as follows: 3 min at 95°C, 10 sec at 95°C, 45 sec at 60°C. The fold change in target gene expression was calculated by the 2^−ΔCt^ relative quantification method and by the 2^−ΔΔCt^ for target gene oscillation. (Applied Biosystems).

### 2.5. Western blot analyses

Cells were homogenized in lysis buffer (pH 7.8, 20 mM Tris, 145 mM NaCl, 5% glycerol, 1% TritonX-100, 50 nM phenylmethylsulfonyl fluoride (PMSF), 50 mM sodium fluoride (NaF), 10 mM sodium orthovanadate (Na3VO4), 50 ng/ml aprotinin, 100 ng/ml leupeptin, 0.8 mg/ml trypsin inhibitor (Sigma, Rehovot, Israel)) as was previously described [35]. Samples were run onto a 10% SDS-polyacrylamide gel. After electrophoresis, proteins were semi-dry-transferred onto nitrocellulose membranes. Blots were incubated with antibodies against AMP-activated protein kinase (AMPK) and its phosphorylated form (pAMPK), acetyl CoA carboxylase (ACC) and its phosphorylated form (pACC), phosphorylated BMAL1 (pBMAL1), AKT (Cell Signalling Technology, Beverly, MA, USA), BMAL1 (Abcam, Cambridge, UK), glycogen synthase kinase 3 beta (GSK3β) and its phosphorylated form (pGSK3β), protein phosphatase 2A (PP2A) and its phosphorylated form (pPP2A), phosphorylated AKT (pAKT) (Santa Cruz Biotechnologies, Santa Cruz, CA, USA), and after several washes, with horseradish peroxidase conjugated secondary antibody (Pierce, Rockford, IL, USA). Anti-mouse antibody (MP Biomedicals, Solon, OH, USA) was used to detect actin, the loading control. The immune reaction was detected by enhanced chemiluminescence (Santa Cruz Biotechnologies, Santa Cruz, CA, USA). Finally, bands were quantified by scanning and densitometry and expressed as arbitrary units.

### 2.6. Statistical analyses

All results are expressed as means ± SE. A one-way ANOVA (time of day) was performed to analyse circadian pattern of clock genes with several time-points. Tukey’s honestly significant difference (HSD) was performed as a single-step multiple comparison procedure in conjunction with ANOVA for the evaluation of significant differences among groups. A student’s t-test was performed for the evaluation of significant differences between two groups. Further analysis of circadian rhythmicity was performed using Circwave software (version 1.4) (Circadian Rhythm Laboratory, University of Groningen, Groningen, Holland). For all analyses, the significance level was set at *p* < 0.05. Statistical analysis was performed with JMP (version 14) software (SAS Institute, Inc., Cary, NC, USA).

## 3. Results

### 3.1. Low doses of palmitate do not lead to fat accumulation

**Figure 1.**
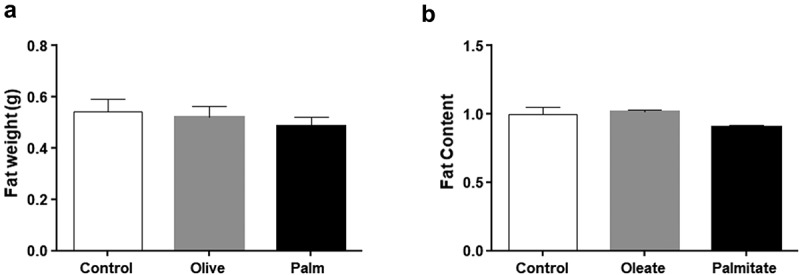
Low doses of fatty acids do not affect fat accumulation in 3T3-L1 adipocytes and mouse WAT. (a) Epididymal WAT weight. B. 3T3-L1 adipocytes lipid content. Mice were fed with palm or olive oil diet for 3 weeks. WAT samples were collected every 4 h for 24 h. Differentiated 3T3-L1 adipocytes were treated with fatty acids for 24 hours. Fat content was measured by oil red staining. Data presented as means ±SE; n = 24/group (for mice), n = 8/group (for cells).

Three weeks of palm or olive oil replacing soybean oil in the regular chow diet of mice did not affect food consumption or body weight [33]. Similarly, no statistical difference in WAT weight was observed (Figure 1(a)). We next tested whether the predominant fatty acids in palm and olive oil, i.e. palmitate and oleate, respectively, affect fat accumulation in cell culture. 3T3-L1 adipocytes treated with low concentrations (50 μM) of palmitate or oleate led to no fat accumulation (Figure 1(b)). These results indicate that low doses of palmitate do not lead to fat accumulation in mouse WAT or 3T3-L1 adipocytes.

### 3.2. Palmitate leads to high-amplitude circadian rhythms

**Figure 2.**
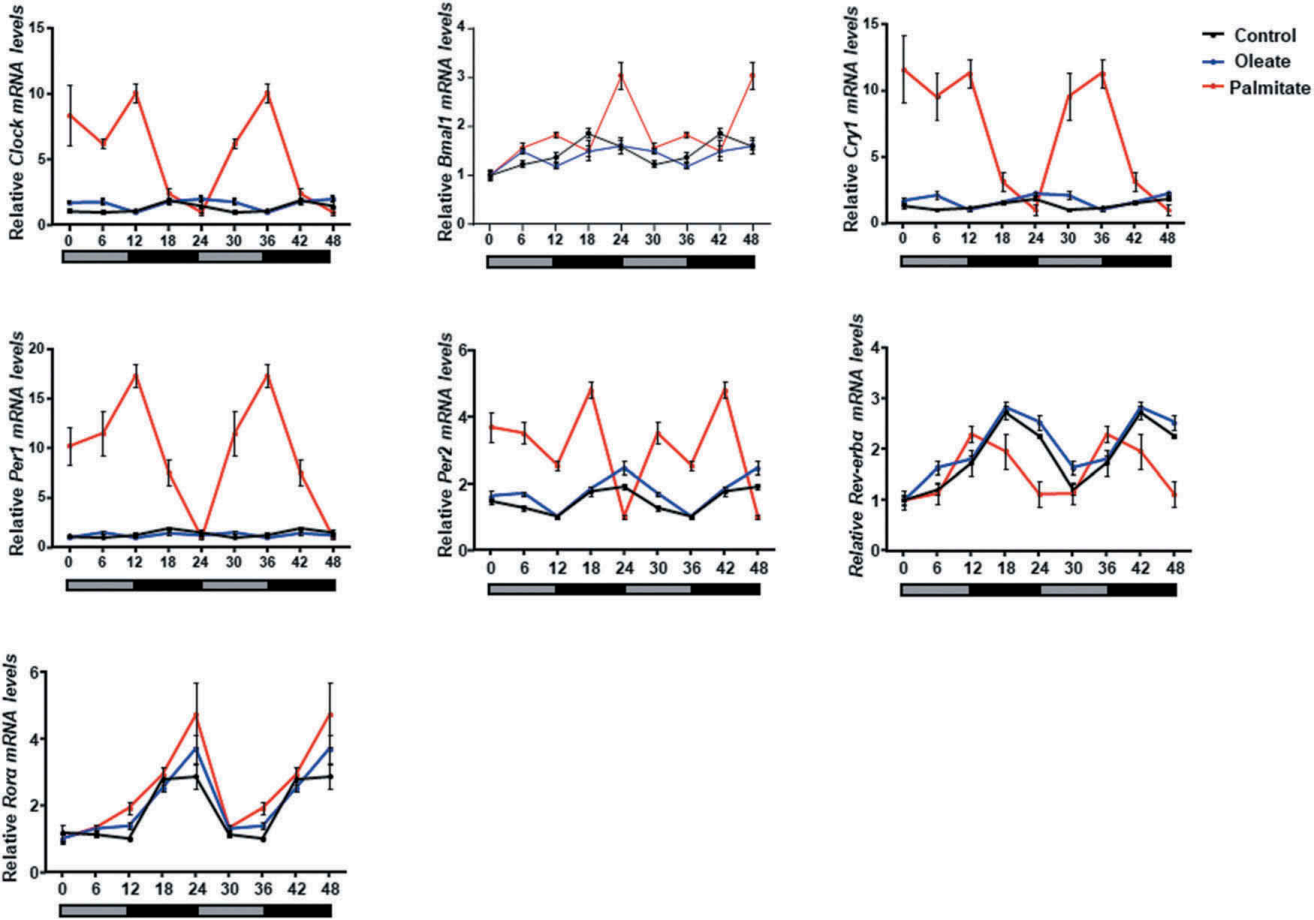
Effect of palmitate and oleate on circadian rhythms. Clock gene oscillations in 3T3-L1 adipocytes. After differentiation, cells were treated with fatty acids. Samples were collected every 6 h for 24 h. Total RNA was extracted, reverse-transcribed and expression levels were determined by real-time PCR. Results are presented as double plotted oscillations. Data are means ± SE; n = 6 for each time-point in each group.

**Figure 3.**
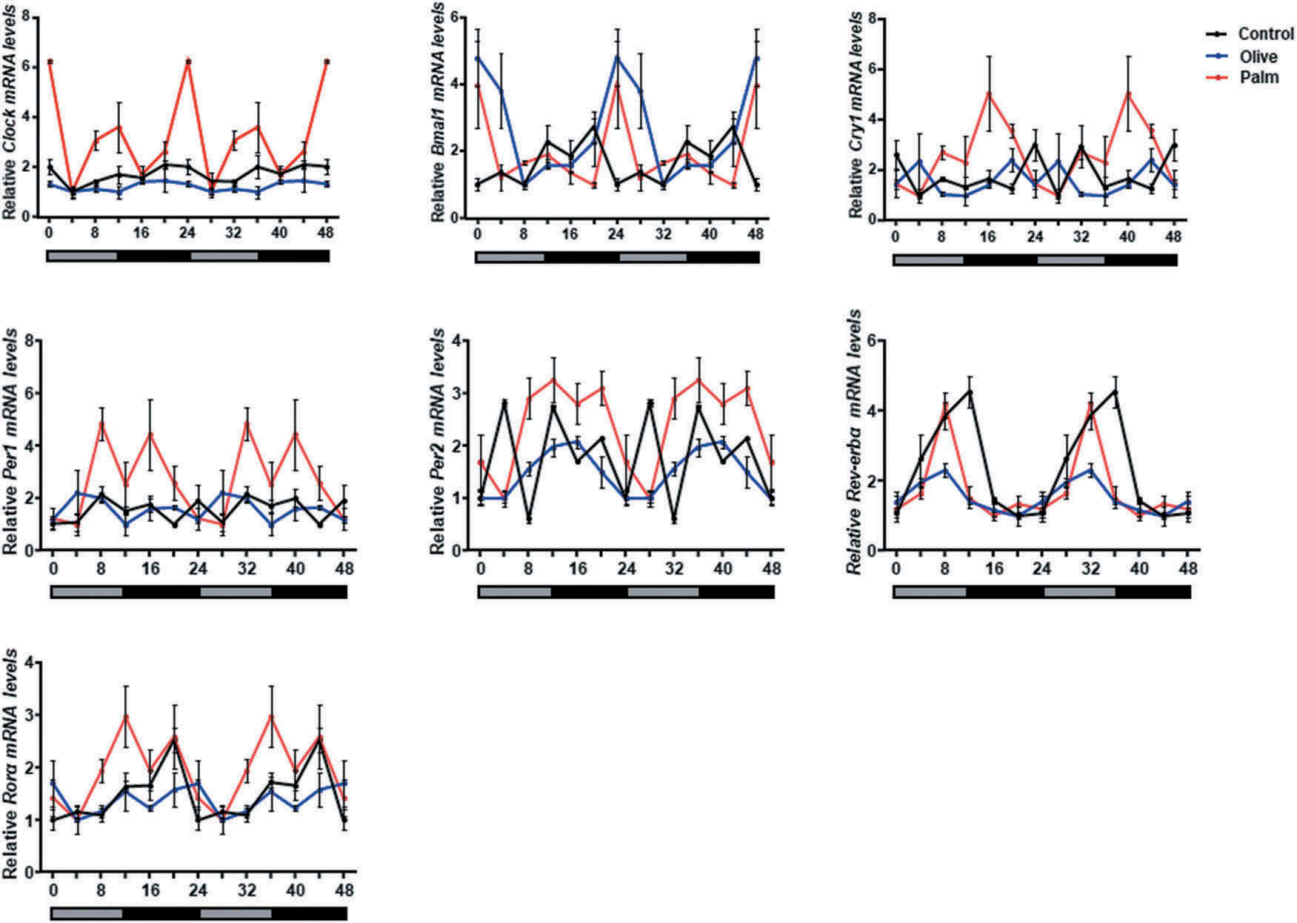
Effect of palm and olive diet on circadian rhythms in mouse epididymal WAT. Mice were fed with palm or olive oil diet for 3 weeks. WAT samples were collected every 4 h for 24 h. Total RNA was extracted, reverse-transcribed and expression levels were determined by real-time PCR. Results are double-plotted oscillations. Data are means ± SE; n = 4 for each time-point in each group.

We next assessed the effect of low concentrations of palmitate and oleate on circadian rhythms. Six time-points throughout the circadian cycle were used to measure circadian gene expression in 3T3-L1 adipocytes. Palmitate, but not oleate, treatment led to increased amplitude of the clock genes *Clock, Bmal1, Cry1, Per1, Per2* and *Rorα* compared to control (Figure 2, Table 1). Increased circadian amplitudes were also seen in mice after 3 weeks of treatment with palm oil, but not olive oil (Figure 3, Table 2). These results indicate that low doses of palmitate are sufficient to alter circadian rhythms in mouse WAT and 3T3-L1 adipocytes.

**Table 1.** Amplitudes of clock genes in 3T3-L1 adipocytes treated with oleate or palmitate. Fold change is relative to control.

	Amplitude			Fold Change	
	BSA	Oleate	Palmitate	Oleate	Palmitate
*Clock*	0.42	0.50	4.53	1.18	10.78
*Bmal1*	0.43	0.31	1.01	0.71	2.36
*Cry1*	0.43	0.61	5.30	1.44	12.43
*Per1*	0.44	0.22	8.18	0.51	18.67
*Per2*	0.45	0.73	1.90	1.60	4.18
*Rorα*	0.93	1.34	1.86	1.44	1.99
*Rev-erbα*	0.86	0.91	0.65	1.05	0.75

**Table 2.** Amplitudes of clock genes in WAT of mice fed soybean, olive or palm oil diet. Fold change is relative to soybean diet (control).

	Amplitude			Fold change	
Gene	Soybean	Olive	Palm	Olive	Palm
*Clock*	0.55	0.24	2.63	0.43	4.78
*Bmal1*	0.87	1.32	0.85	1.52	0.98
*Cry1*	1.01	0.70	5.76	0.69	5.69
*Per1*	0.58	0.60	1.91	1.04	3.30
*Per2*	0.91	0.54	1.12	0.59	1.24
*Rorα*	0.76	0.35	0.98	0.46	1.29
*Rev-erbα*	1.77	0.65	1.58	0.37	0.89

### 3.3. Palmitate downregulates the expression of circadian clock

**Figure 4.**
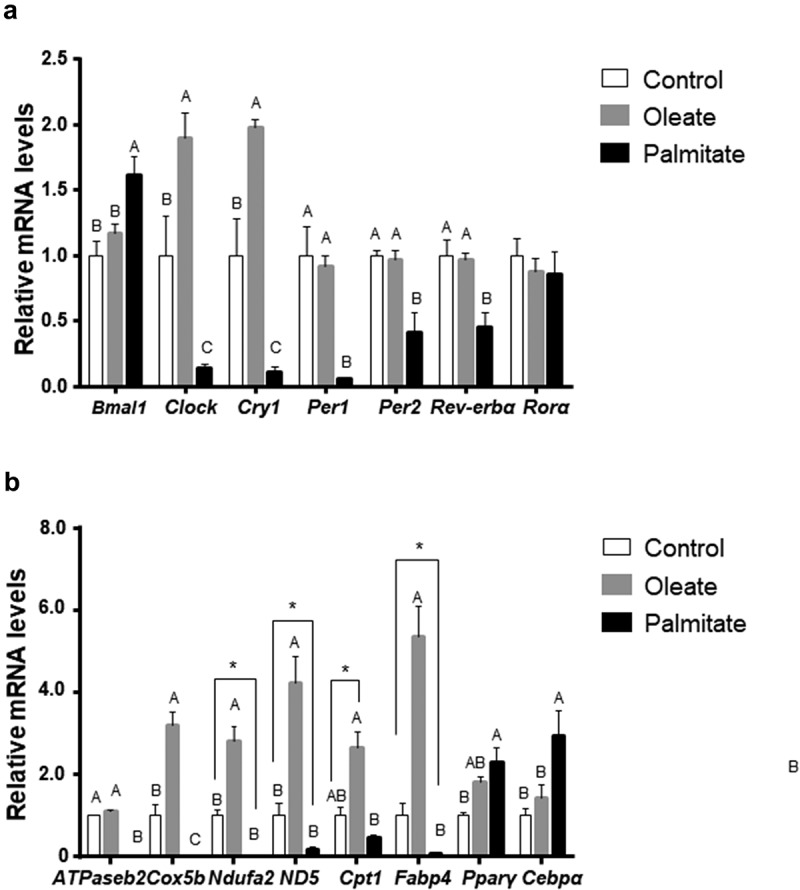
Effect of palmitate and oleate on circadian and metabolic genes. Differentiated 3T3-L1 adipocytes were treated with fatty acids for 24 h. Total RNA was extracted, reverse-transcribed and expression levels were determined by real-time PCR. (a) mRNA levels of the circadian clock genes *Clock, Bmal1, Cry1, Per1, Per2, Rev-erbα* and *Rorα*. (b) mRNA levels of the metabolic genes *ATPase2b, Cox5b, Ndufa2, Nd5, Cpt1, Fabp4, Pparγ* and *Cebpα*. Data are mean ± SE; n = 6. Different letters denote significant difference (p < 0.05, Tukey’s HSD), asterisk denotes significant difference (p < 0.05, Student’s t-test).

As high amplitude rhythms do not always reflect higher expression level, we next measured clock mRNA levels after 24 h in 3T3-L1. Twenty-four hours after treatment, palmitate significantly decreased the mRNA levels of *Clock* (p = 0.026, Tukey’s HSD), *Cry1* (p = 0.0049, Tukey’s HSD), *Per1* (p = 0.0005, Tukey’s HSD), *Per2* (p = 0.0026, Tukey’s HSD) and *Rev-erbα* (p = 0.0035, Tukey’s HSD) (Figure 4(a)). In contrast, palmitate led to increased *Bmal1* mRNA levels (p = 0.0037, Tukey’s HSD). Oleate treatment led to increased *Clock* (p = 0.0209, Tukey’s HSD) and *Cry1* (p = 0.0024, Tukey’s HSD) mRNA levels (Figure 4(a)). These results indicate that treatment with palmitate decreases the expression of the circadian clock.

### 3.4. Palmitate modulates circadian metabolism

As the circadian clock controls metabolism, we next measured the expression level of metabolic genes 24 h after treatment with low concentrations (50μM) of palmitate or oleate. Palmitate upregulated the adipogenic genes *Pparγ* (p = 0.0028, Tukey’s HSD) and *Cebpα* (p = 0.0084, Tukey’s HSD) and downregulated *Fabp4* levels (p = 0.0249, Student’s t-test) and the mitochondrial genes *Cox5b* (p = 0.031, Tukey’s HSD), *ATPaseb2*, (p ≤ 0.001, Tukey’s HSD) *Ndufa2* (p = 0.0057, Student’s t-test) and *ND5* (p = 0.0393, Student’s t-test). In contrast, oleate significantly increased *FABP4* mRNA levels (p ≤ 0.001, Tukey’s HSD) and the mitochondrial genes *Cox5b* (p ≤ 0.001, Tukey’s HSD), *Ndufa2* (p = 0.0022, Tukey’s HSD), *ND5* (p = 0.0001, Tukey’s HSD and *Cpt1* (p = 0.04, Student’s t-test). (Figure 4(b)). These results suggest that treatment with palmitate modulates lipid metabolism by increasing adipogenesis and lipogenesis and decreasing mitochondrial electron transport chain and ATP synthesis, while treatment with oleate increases the expression of genes involved in catabolism and ATP generation.

### 3.5. Palmitate disrupts catabolic pathways

**Figure 5.**
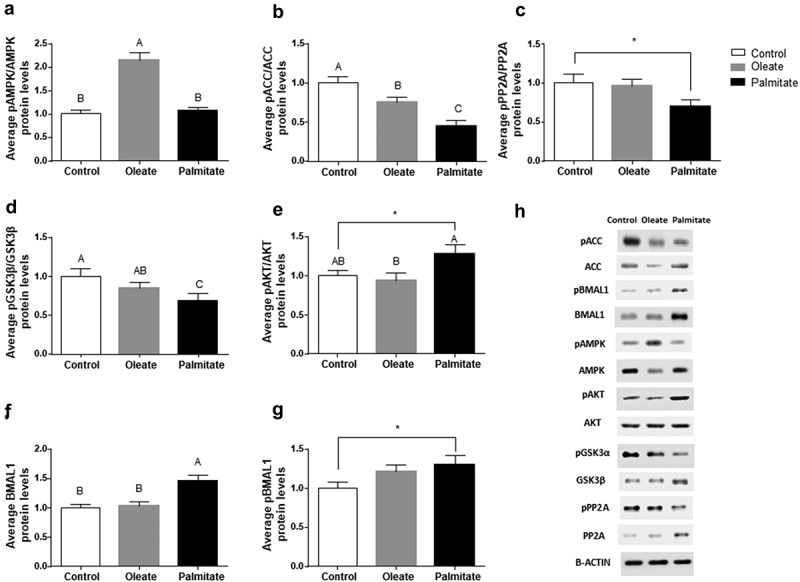
Effect of palmitate on metabolic pathways. Differentiated 3T3-L1 adipocytes were treated with fatty acids. Protein samples were collected every 6 h for 24 h and analysed separately by Western blot. (a) Daily average protein levels of pAMPK/AMPK ratio. (b) Daily average protein levels of pACC/ACC ratio. (c) Daily average protein levels of pPP2A/PP2A ratio. (d) Daily average protein levels of pGSK3β/GSK3β ratio. (e) Daily average protein levels of pAKT/AKT ratio. (f) Daily average protein levels of BMAL1. (g) Daily average protein levels of pBMAL1. Data presented as means ± SE; n = 30/group, n = 6/time-point. Different letters denote significant difference (p ≤ 0.05, Tukey’s HSD), asterisk denotes significant difference (p ≤ 0.05, Student’s t-test).

To elucidate the metabolic pathways modulated by palmitate and oleate, we next assessed the daily average expression and/or activation levels of the key metabolic regulators AMPK, ACC, PP2A, GSK3β and AKT in 3T3-L1 adipocytes. Palmitate did not affect, while oleate significantly increased, the pAMPK/AMPK ratio compared to control (p < 0.001, Tukey’s HSD) (Figure 5(a)). In contrast, palmitate as well as oleate significantly increased ACC activity (low pACC/ACC ratio) compared to control (p < 0.001, Tukey’s HSD) (Figure 5(b)). To elucidate why the increase in ACC activity was not mirrored by reduced AMPK activity (low pAMPK/AMPK), we measured the average levels of PP2A, the active form of the phosphatase involved in ACC dephosphorylation. Palmitate significantly reduced the pPP2A/PP2A ratio compared to control (p = 0.04, Student’s t-test) (Figure 5(c)) suggesting increased dephosphorylation of pACC and, as a result, increased ACC activity. PP2A is also involved in the activation of the metabolic regulator GSK3β by dephosphorylating it [36]. Therefore, we measured GSK3β levels following palmitate treatment. Palmitate significantly reduced the pGSK3β/GSK3β ratio (p = 0.0479, Tukey’s HSD) indicating activation of GSK3β by palmitate (Figure 5(d)). In addition, palmitate led to a higher activation of AKT (Figure 5(e)). As GSK3β regulates BMAL1 [20], we next measured BMAL1 levels. Palmitate significantly increased BMAL1 (p < 0.001, Tukey’s HSD) and pBMAL1 levels (p = 0.0268, Student’s t-test) compared to control (figure 5(f,g)).

## 4. Discussion

In this study, we demonstrated for the first time the effect of palmitate and oleate on the circadian clock in 3T3-L1 adipocytes. We show that non-obesogenic doses of palmitate were sufficient to alter circadian rhythms by increasing clock gene amplitudes both *in vivo*, in mice fed palm oil diet, and in cell culture, in 3T3-L1 adipocytes, with no effect of oleate. This finding is congruent with the reported effect of palmitate in mHypo-37 neuronal cells, inducing increased amplitude of *Bmal1*, which was lowered in the presence of DHA [29]. We have recently shown that low doses of palmitate altered circadian rhythms in mouse liver and AML-12 hepatocytes by delaying the phase of clock gene expression [33]. Although palmitate treatment increased clock gene amplitudes, it decreased the expression of the circadian clock by downregulating the mRNA levels of the clock genes *Clock, Cry1, Per1, Per2* and *Rev-erbα*. Such observation is concordant with a recent report showing that palmitate suppressed clock gene expression [30]. In contrast to the suppression of these clock genes, palmitate led to increased *Bmal1* mRNA and protein levels.

BMAL1 has been reported to regulate lipid accumulation in adipose tissue [2,12]. Its increased expression, as a result of palmitate treatment, was mirrored by a high expression of adipogenic markers, including *Pparγ* and *Cebpα*. Moreover, palmitate decreased the expression of the gene encoding the fatty acid chaperone FABP4, that triggers the ubiquitination and subsequent proteasomal degradation of PPARγ [37]. In addition, palmitate reduced the expression of genes encoding proteins involved in mitochondrial electron transport chain and ATP synthesis, such as *Atpase2b, Cox5b, Ndufa2*, and *Nd5*. In contrast, oleate induced the expression of the gene encoding FABP4, the expression of genes encoding the aforementioned electron transport chain and ATP synthesis proteins and *Cpt1* mRNA levels. Thus, palmitate increases signalling towards lipogenesis and decreases signalling towards fatty acid oxidation, while oleate increases catabolic signalling pathways. These metabolic findings are concordant with our previous report showing the effect of oleate and palmitate on mouse liver and hepatocytes [33].

AMPK is a key metabolic factor that regulates circadian clock [2]. While palmitate did not affect AMPK activation, oleate increased AMPK activation, similarly to previous reports [33,38,39]. GSK3β, another key metabolic factor has been shown to phosphorylate most clock proteins, thereby controlling their stability and subcellular localization [19]. GSK3β phosphorylates BMAL1 and primes it for ubiquitination. In the absence of GSK3β-mediated phosphorylation, BMAL1-dependent circadian gene expression is dampened [20]. Our results with palmitate are in congruent with these findings, as palmitate induced GSK3β activation, increased phosphorylated BMAL1 levels and clock amplitudes. In addition, palmitate induced activation of AKT. Recent reports showed a positive feedback of GSK3β on AKT [40–42]. AKT also phosphorylates BMAL1. AKT-mediated phosphorylation of BMAL1 stabilizes BMAL1 and induces its dissociation from DNA and subsequently nuclear exclusion, which results in the suppression of BMAL1 transcriptional activity [43]. This is congruent with our findings that palmitate activated AKT, increased BMAL1 and phosphorylated BMAL1 protein levels and downregulated clock gene expression level.

In summary, in adipocytes, oleate activates the AMPK signalling pathway leading to inhibition of fatty acid synthesis and increased fatty acid oxidation. In contrast, palmitate activates ACC pathway leading to increased fatty acid synthesis. This is achieved by inducing the activity of PP2A. Induced PP2A activity increases GSK3β activity and, as a result AKT activity, leading to the phosphorylation of BMAL1, which leads to adipogenesis and lipogenesis and high amplitude but dampened clock gene expression. The intricate relationship between core clock proteins and fatty acids warrants further study.
