# Supplementary material for: Non-obesogenic doses of palmitate disrupt circadian metabolism in adipocytes
Source: Adipocyte. 2019 Dec 3;8(1):392–400. doi: 10.1080/21623945.2019.1698791 (PMC6948973; doi:10.1080/21623945.2019.1698791)
Supplement: Supplemental Material [file kadi-08-01-1698791-s001.zip › Tal et al., Supplementary Figures S1-S2.pptx]

## Slide 1
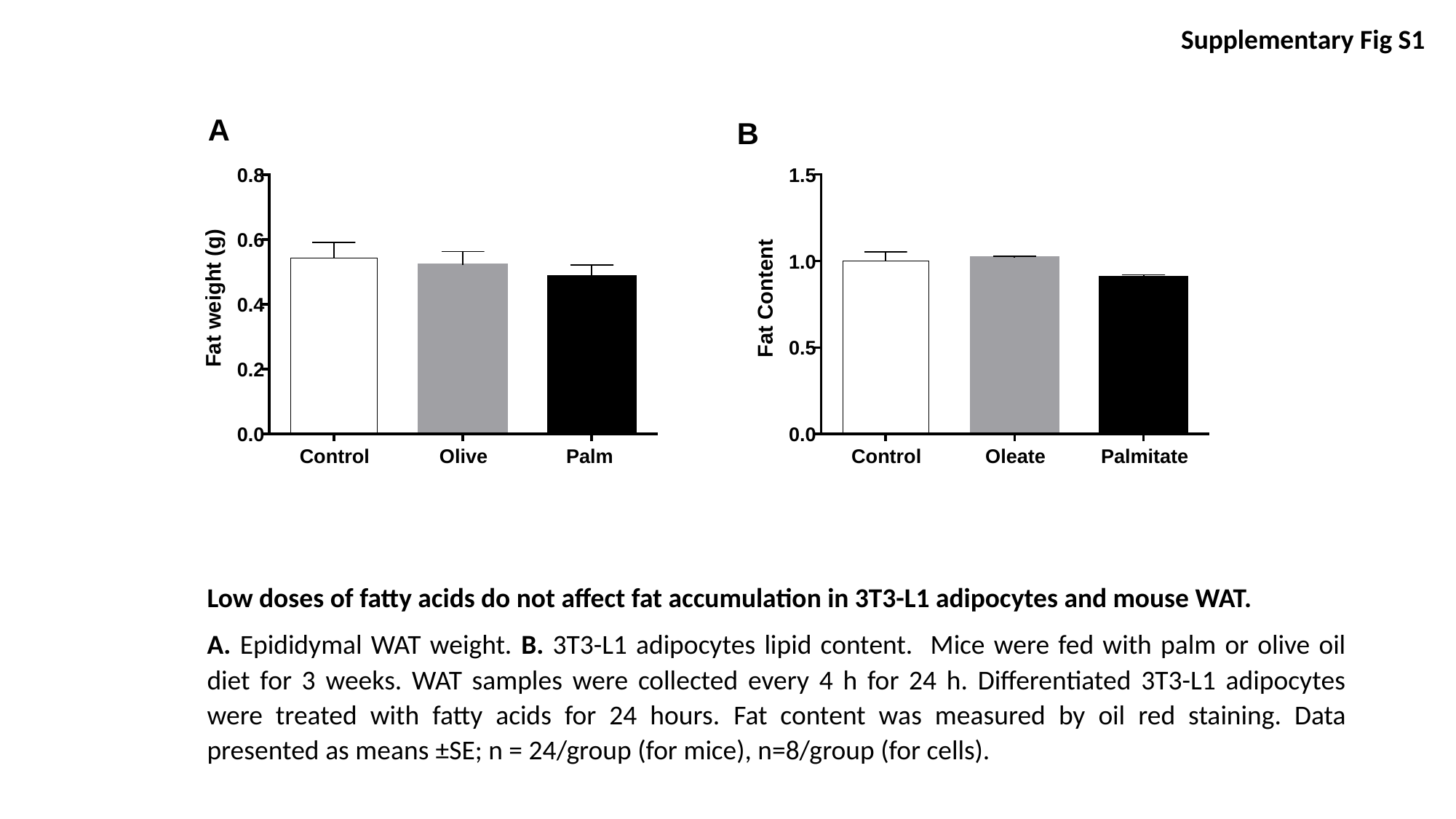

Supplementary Fig S1
A
B
0.8
0.6
Fat weight (g)
0.4
0.2
0.0
Control
Olive
Palm
1.5
1.0
Fat Content
0.5
0.0
Control
Oleate
Palmitate
Low doses of fatty acids do not affect fat accumulation in 3T3-L1 adipocytes and mouse WAT.
A. Epididymal WAT weight. B. 3T3-L1 adipocytes lipid content. Mice were fed with palm or olive oil diet for 3 weeks. WAT samples were collected every 4 h for 24 h. Differentiated 3T3-L1 adipocytes were treated with fatty acids for 24 hours. Fat content was measured by oil red staining. Data presented as means ±SE; n = 24/group (for mice), n=8/group (for cells).

## Slide 2
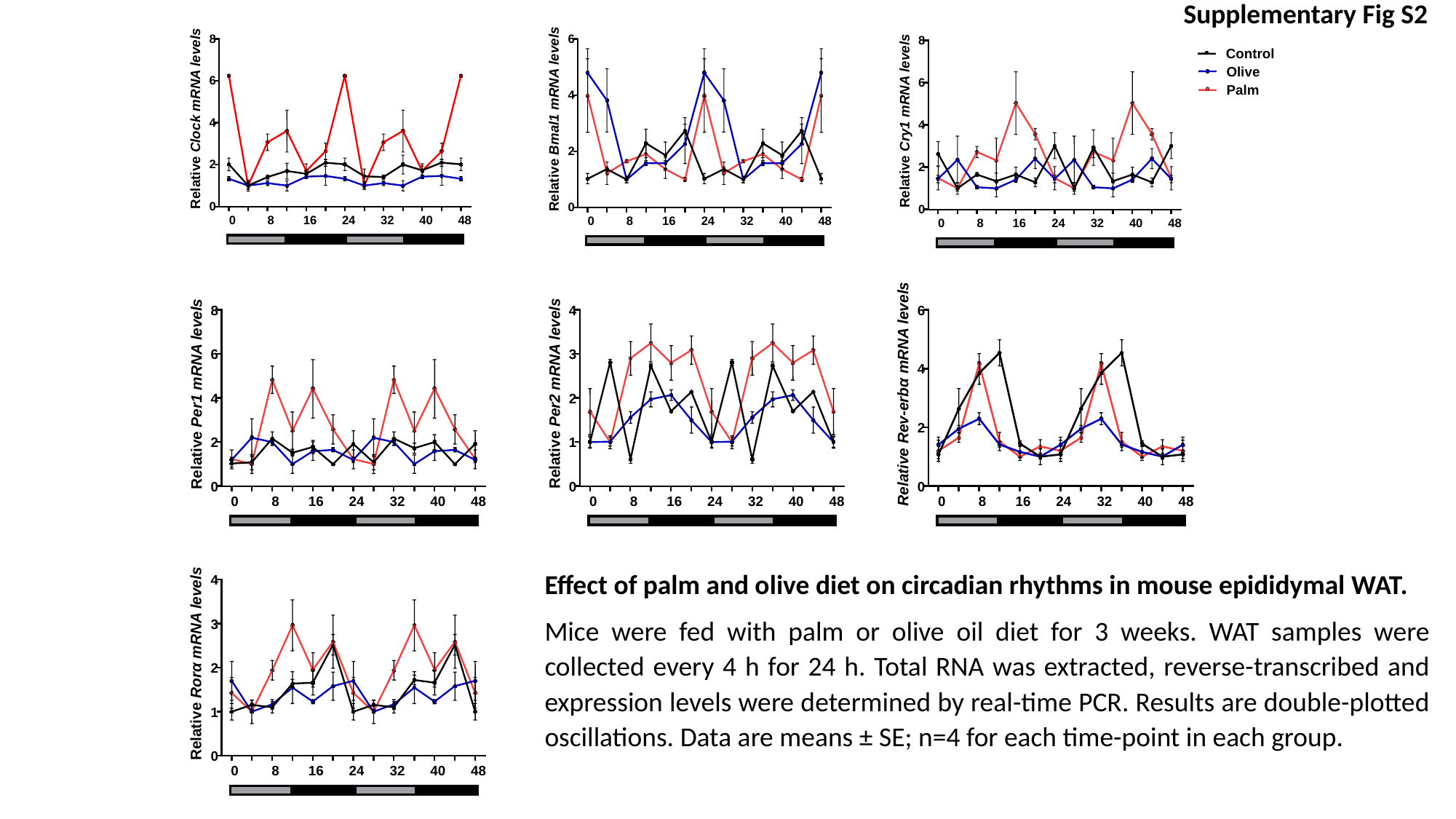

6
4
Relative Bmal1 mRNA levels
2
0
0
8
16
24
32
40
48
8
6
Relative Clock mRNA levels
4
2
0
0
8
16
24
32
40
48
8
6
Relative Cry1 mRNA levels
4
2
0
0
8
16
24
32
40
48
Control
Olive
Palm
6
4
Relative Rev-erbα mRNA levels
2
0
0
8
16
24
32
40
48
4
3
Relative Per2 mRNA levels
2
1
0
0
8
16
24
32
40
48
8
6
Relative Per1 mRNA levels
4
2
0
0
8
16
24
32
40
48
4
3
Relative Rorα mRNA levels
2
1
0
0
8
16
24
32
40
48
Supplementary Fig S2
Effect of palm and olive diet on circadian rhythms in mouse epididymal WAT.
Mice were fed with palm or olive oil diet for 3 weeks. WAT samples were collected every 4 h for 24 h. Total RNA was extracted, reverse-transcribed and expression levels were determined by real-time PCR. Results are double-plotted oscillations. Data are means ± SE; n=4 for each time-point in each group.
