# Supplementary material for: Non-obesogenic doses of palmitate disrupt circadian metabolism in adipocytes
Source: Adipocyte. 2019 Dec 3;8(1):392–400. doi: 10.1080/21623945.2019.1698791 (PMC6948973; doi:10.1080/21623945.2019.1698791)
Supplement: Supplemental Material [file kadi-08-01-1698791-s001.zip › Tal et al., Supplementary Table S1.docx]

**Table S1: Primers used in RT-PCR**

| **Gene Forward Reverse** | | |
| --- | --- | --- |
| *Actin* | 5’-CTAAGGCCAACCGTGAAAAG-3’ | 5’-GGGGTGTTGAAGGTCTCAAA-3’ |
| *Clock* | 5'-CCTAGAAAATCTGGCAAAATGTCA-3' | 5'-CCTTTTCCATATTGCATTAAGTGCT-3' |
| *Bmal1* | 5'-CAAGAATGCAAGGGAGGCC-3' | 5'-TTGTCCCGACGCCTCTTTT-3' |
| *Cry1* | 5'-AGCCAGCTGATGTATTTCCCA-3' | 5'-AGTTTAGTGATGTTCCATTCCTTGAA-3' |
| *Per1* | 5'-CCGAATACACACTTCGAAACCAG-3' | 5'-TCCCGTTTGCAACGCAG-3' |
| *Per2* | 5’-CGGGCTATGAAGCGCCTAG-3’ | 5’-GGTTGTTGTGAAGATCCTCTTCTCA-3’ |
| *Rorα* | 5-CAGAAATGCCTGGCCGTG-3’ | 5’-CCGACCAAACTTGACAGCATC-3’ |
| *Rev-erbα* | 5’- GGGCACAAGCAACATTACCAA-3’ | 5’-CACCTTACACAGTAGCACCATGC-3’ |
| *Pparγ* | 5’-CACAATGCCATCAGGTTT-3’ | 5’-CAGCTTCTCCTTCTCGGC-3’ |
| *Cebpα* | 5’-AGTACCGGGTACGGCGGGAAC -3’ | 5’-GCGTGTCCAGTTCACGGCTCA -3’ |
| *Fabp4* | 5’-GAAAACGAGATGGTGACAAGC -3’ | 5’-TTGTGGAAGTCACGCCTTT -3’ |
| *ATPaseb2* | 5’-ACCTATCCCAGCCTCGTC -3’ | 5’-AGGACTTGCCCACTTCTCTTT -3’ |
| *Cox5b* | 5'- GCTGCATCTGTGAAGAGGACAAC -3' | 5'- CAGCTTGTAATGGGTTCCACAGT -3' |
| *Ndufa2* | 5'-AGCCTGAAGGTCTCCACTGA -3' | 5'CAGTGTTGCGCAGTAAGAGG- -3' |
| *ND5* | 5'- AGCATTCGGAAGCATCTTTG -3' | 5'- TTGTGAGGACTGGAATGCTG -3' |
