# Supplementary material for: Non-obesogenic doses of palmitate disrupt circadian metabolism in adipocytes
Source: Adipocyte. 2019 Dec 3;8(1):392–400. doi: 10.1080/21623945.2019.1698791 (PMC6948973; doi:10.1080/21623945.2019.1698791)
Supplement: Supplemental Material [file kadi-08-01-1698791-s001.zip › Tal et al., Supplementary Table S2.docx]

**Supplementary Table S2: Amplitudes of clock genes in WAT of mice fed soybean, olive or palm oil diet. Fold change is relative to soybean diet (control).**

|  | \| **Amplitude** \| \| --- \| \|  \| | | | \| **Fold change** \| \| --- \| \|  \| | |
| --- | --- | --- | --- | --- | --- | --- | --- | --- | --- |
| **Gene** | **Soybean** | **Olive** | **Palm** | **Olive** | **Palm** |
|  |  |  |  |  |  |
| ***Clock*** | **0.55** | **0.24** | **2.63** | **0.43** | **4.78** |
| ***Bmal1*** | **0.87** | **1.32** | **0.85** | **1.52** | **0.98** |
| ***Cry1*** | **1.01** | **0.70** | **5.76** | **0.69** | **5.69** |
| ***Per1*** | **0.58** | **0.60** | **1.91** | **1.04** | **3.30** |
| ***Per2*** | **0.91** | **0.54** | **1.12** | **0.59** | **1.24** |
| ***Rorα*** | **0.76** | **0.35** | **0.98** | **0.46** | **1.29** |
| ***Rev-erbα*** | **1.77** | **0.65** | **1.58** | **0.37** | **0.89** |
